# Does IFN-γ play a role on the pathogenesis of non-atopic asthma in Latin America children?

**DOI:** 10.1186/1710-1492-8-18

**Published:** 2012-12-19

**Authors:** Camila Alexandrina Figueiredo, Laura Cunha Rodrigues, Neuza Maria Alcantara-Neves, Philip J Cooper, Leila Denise Amorim, Nivea Bispo Silva, Alvaro A Cruz, Mauricio Lima Barreto

**Affiliations:** 1 Instituto de Ciências da Saúde, Universidade Federal da Bahia, Salvador, Brazil; 2 London School of Hygiene and Tropical Medicine, London, UK; 3 Molecular and Biochemical Parasitology, Liverpool School of Tropical Medicine, Liverpool, UK; 4 Colegio de Ciencias de La Salud, Universidad San Francisco de Quito, Quito, Ecuador; 5 Instituto de Matemática, Universidade Federal da Bahia, Salvador, Brazil; 6 ProAR – Núcleo de Excelência em Asma, Universidade Federal da Bahia and CNPq, Salvador, Brazil; 7 Instituto de Saúde Coletiva, Universidade Federal de Bahia, Rua Basilio da Gama s/n, Canela, 41110-040, Salvador-Bahia, Brazil

**Keywords:** Non-atopic asthma, Cytokines, IFN-g, Monocytes, Atopic-asthma, IgE, Atopy

## Abstract

In this work we explore differences in blood cells and cytokine profiles in children according to atopic status and asthma (atopic or non-atopic). The study involved measurement of Th1(IFN-γ) and Th2 (IL-5 and IL-13) cytokines in * Dermatophagoides pteronyssinus * stimulated peripheral blood leukocytes, blood cell count, skin prick test and specific IgE against common aeroallergens. Atopic status was associated with eosinophilia and production of Th2 type cytokines. Atopic asthma was associated with eosinophilia and non-atopic asthma was associated with IFN-γ and elevated monocytes in blood. IFN-γ and monocytes might play a role in immunopathology of non-atopic asthma in Latin American children.

## Findings

Historically, atopy is associated to asthma especially in developed countries, however, in fact less than half asthma cases worldwide are attributable to atopy, with the population attributable fraction varying from 41% in ‘affluent’ countries to 20% or less in ‘non-affluent’ countries [1]. In this last group are situated Latin America countries [2,3]. Although the immunopathological features of atopic asthma are well characterized as an eosinophilic bronchitis in the airways, with the inflammatory process governed by Th2 cytokines, such as IL-4, IL-5 and IL-13, the immunological and cellular profiles of non-atopic asthma are not well known. Experimental animal studies have shown that Th1 cells rather than counterbalancing Th2-mediated effects may worsen airway inflammation. Similarly, studies of asthma in humans suggest possible roles for interferon-gamma (IFN-γ): a) asthma has been associated with elevated production of IFN-γ but not IL-4 by bronchoalveolar lavage cells [4]; b) greater frequencies of peripheral blood CD8+ T cells expressing IFN-γ in asthmatic airways, correlating with asthma severity and bronchial hyperresponsiveness [5]; c) induced sputum from patients with atopic or non-atopic asthma presented increased eosinophils and IFN-γ [6]. In the present study, we compared the cytokine profile of peripheral blood leukocytes and blood cells counts by asthma phenotype and atopic status in children.

The methods used have been reported in detail elsewhere [7]. This study was conducted in the city of Salvador, BA, Brazil with nearly 2,800,000 inhabitants, mostly of mixed African descent, located in Northeast Brazil. Briefly, we studied 1,445 children aged 4-11 years old, enrolled in a cohort recruited from 1997 and 2003 for evaluating the impact of a sanitation program on the incidence of childhood diarrhea, in different city areas, selected to represent the population without sanitation at that time. 51.7% (n=651) of the kids enrolled were from families having mensal income equal or less than 147 USD in 2005 and only 3.3% have equal or more than 500 USD, characterizing this population as a typical urban poor population. In 2005, these children were resurveyed and where was collected information on wheezing status, serum for IgE, skin prick test, blood cell count and whole blood culture. Analyses regarding cytokines were performed with 788 children who had information on both cytokine response for *Dermatophagoides* and allergy markers. Children were classified into 4 groups based on the detection of allergen-specific IgE in serum (sIgE) and asthma symptoms: atopic asthmatic, non-atopic asthmatic, atopic non-asthmatic and non-atopic non-asthmatic. The prevalence of atopic asthma is 10.9% and non-atopic asthma is 11.3%, while disease severity, based on reported symptoms (such as number of episodes in the last 12 months, difficulty of speech during crises and awaking up at night due to wheezing), is observed among 51.18% atopic asthmatics and 55.86% non-atopic asthmatics. Using hospitalisation as parameter of asthma severity we got 12.6% for atopic asthmatic and 11.03% for non-atopic asthmatic. No great difference on disease severity between atopic asthmatic and non-atopic asthmatic was observed considering both parameters. Atopy was also defined based on SPT reactivity to common aeroallergens. SPTs were performed with extracts of *Dermatophagoides pteronyssinus, Blomia tropicalis, Blattela germanica, Periplaneta americana*, dog and cat epithelia, and a fungi mix (ALK-ABELLO, São Paulo, Brazil). Children were considered positive if the mean diameter of the wheal was ≥3mm after subtraction of the negative control. sIgE for *D pteronyssinus, B tropicalis, P americana*, and *B germanica* in serum was measured according to the manufacturer’s instructions (Phadia Diagnostics AB, Uppsala Sweden). sIgE measurement of ≥0.70 kU/L for at least one of the tested allergens was considered positive. Specifically for *D. pteronyssinus,* 21.4% of 788 children were sensitized to this mite. For the cytokine evaluation, we collected venous blood into heparinized tubes and cultured the blood at a dilution of 1:4 in RPMI (Gibco, Auckland, New Zealand) containing 10 mM glutamine (Sigma- Aldrich, St. Louis, MO, USA) and 100 μg/mL gentamicin (Sigma-Aldrich, St. Louis, MO, USA). The cell cultures were set up within 6 h of blood collection and were maintained in a humidified environment of 5% CO2 at 37°C for 5 days in the presence of endotoxin-free *D. pteronyssinus* antigen (ALK-ABELLO) (5 μg/mL) or media alone for the detection of IL-13, IL-5, and IFN-γ by ELISA (BD Pharmingen San Diego, CA, USA). Children with cytokine concentrations above the lower detection limits after subtracting negative control values were classified as responders.

The International Study of Asthma and Allergies in Childhood (ISAAC) questionnaire, translated into Brazilian Portuguese [8], was applied to the child’s parents. Asthma was defined as wheezing in the previous 12 months and at least one of the following: diagnosis of asthma at least once in life or waking up at night because of wheezing, wheezing while exercising or four or more episodes of wheezing the past 12 months. Associations between cytokine responsiveness with atopic status and the four asthma groups were assessed using the Pearson chi-squared test adjusted for gender and age. Fisher’s exact test was also used when appropriate. Geometric means (GM) and 95% confidence intervals were obtained for analysis of differential blood cell count. Mann-Whitney and Kruskal-Wallis tests were conducted to compare blood cell counts across the groups. Logistic regression model was used for evaluation of the simultaneous effect of differential blood cells count and Th2 cytokines on asthma.

**Figure 1:**
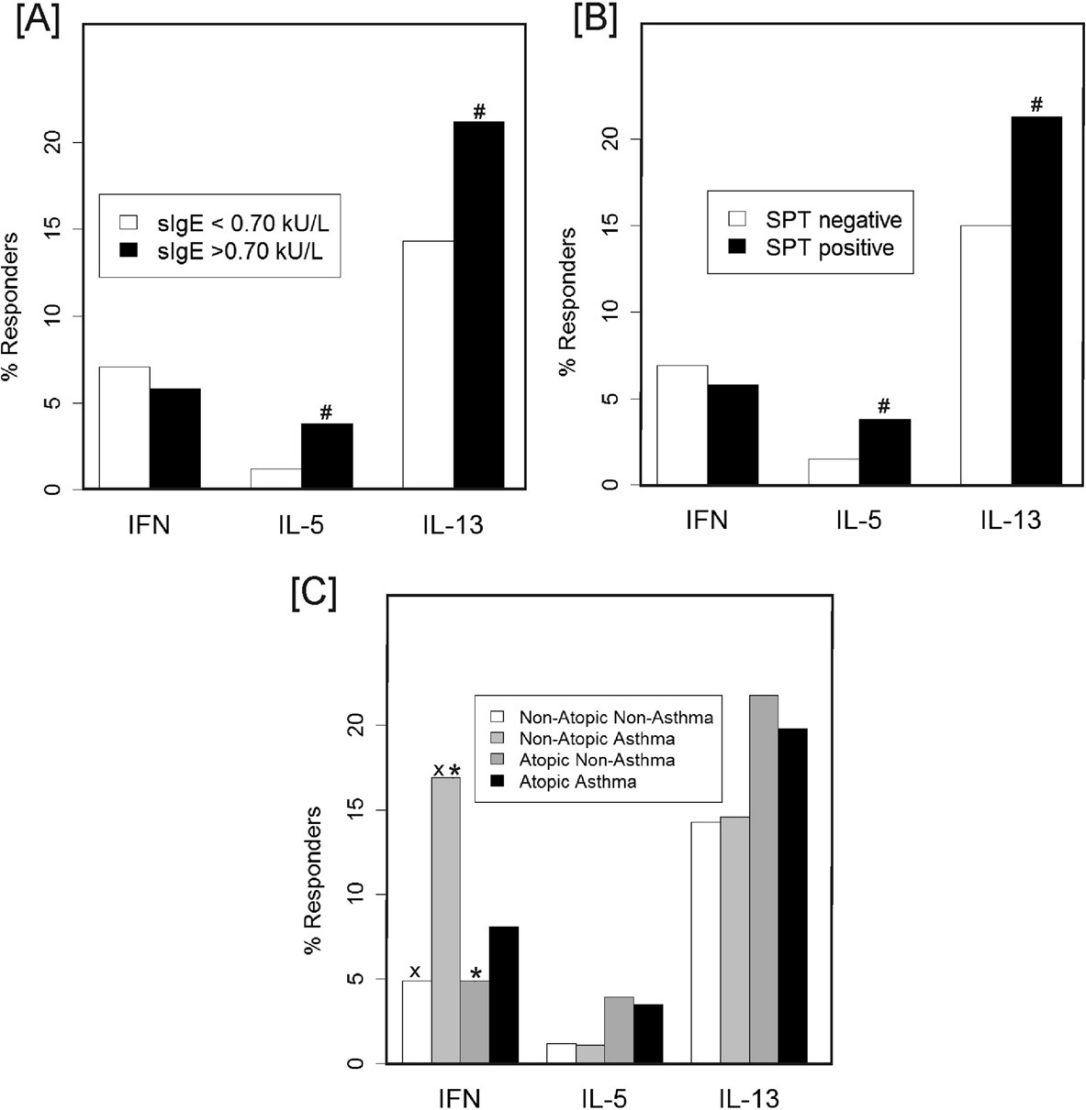
Cytokine profile of* Dermatophagoides pteronyssinus *stimulated PBLs by levels of IgE (A), and skin test reactivity (SPT) to common aeroallergens (B); and by asthma phenotypes (C) (# p<0.05 adjusted for gender and age; × and * p<0.001 for multiple comparisons).

**Figure 2:**
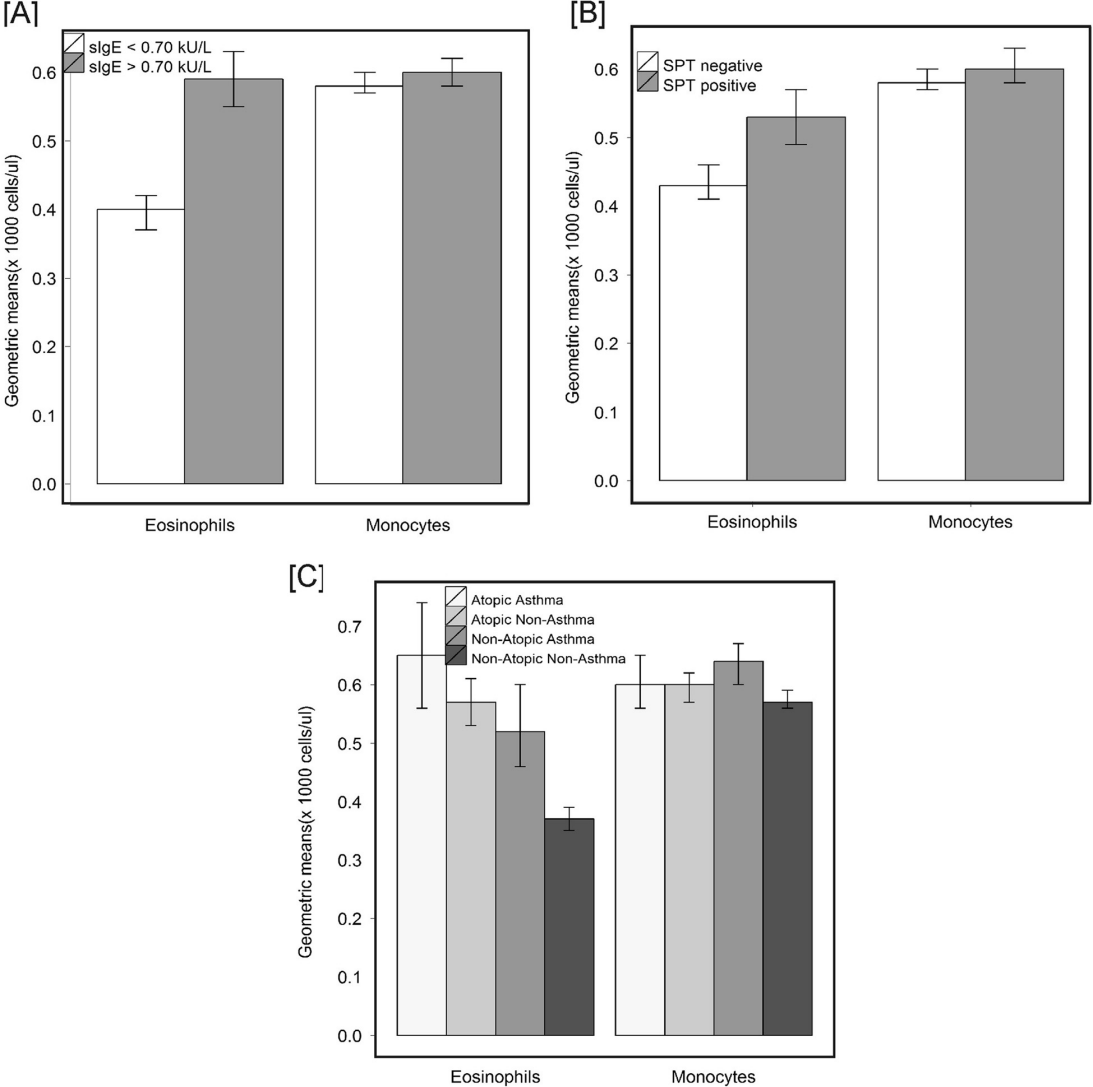
Geometric means and 95% confidence intervals of eosinophil and monocytes in peripheral blood by presence of allergen-specific IgE (sIgE) (A), allergen skin test reactivity (SPT) (B) and asthma phenotypes (C).

The proportions of children producing detectable levels of Th2 cytokines were greater among children with a positive sIgE in comparison to non-sensitized children (IL-5: 3.8% vs 1.2% p=0.018; IL-13: 21.2% vs 14.3% p=0.032) (Figure 1a). The proportions of Th2 cytokines were also higher in children with SPT reactivity in comparison to SPT negative children (IL-5: 3.8% vs 1.5% p=0.042; IL-13: 21.3% vs 15% p=0.036) (Figure 1b). The proportion of children producing detectable levels of IFN-γ was higher among non-atopic asthmatics compared to atopic non-asthmatics (16.9% vs 4.9%, p=0.0013) and non-atopic non-asthmatics (16.9% vs 4.9%, p=0.0002) (Figure 1c). Children with detectable sIgE or SPT reactivity had greater counts of blood leukocyte cells, although this was significant only for sIgE (Mann-Whitney test *P*=0.008–data not shown). In addition, eosinophil counts in peripheral blood were greater in children with a positive sIgE (0.59x10^3^cels/ml (95%CI= 0.55;0.63) vs. 0.40x10^3^cels/ml (95%CI=0.37;0.42)) (Figure 2a) or SPT reactivity (0.53x10^3^cels/ml (95%CI=0.49;0.57) vs. 0.43x10^3^cels/ml (95%CI=0.41;0.46)) (Figure 2b) both in comparison to non-sensitized kids. Children with non-atopic asthma had higher monocyte counts than non-atopic non-asthmatics (0.64x10^3^cels/ml (95%CI=0.60;0.67) vs 0.57x10^3^cels/ml (95%CI=0.56;0.59)) (Figure 2c). Non-atopic non-asthmatics had lower eosinophil counts (0.37x10^3^cels/ml (95%CI=0.35; 0.39) compared to non-atopic asthmatics (0.52x10^3^cels/ml (95%CI=0.46;0.60) and atopic non-asthmatics (0.57x10^3^cels/ml (95%CI=0.53;0.61)) (Figure 2c). Lymphocyte and neutrophil counts were similar in the 4 study groups (data not shown). Through the logistic regression model we observed that eosinophils are significantly associated to the occurrence of atopic asthma even after controlling for Th2 cytokines production, indicating that Th2 cytokines may play a role on increasing eosinophil inflammation on atopic asthma (data not shown).

We have shown that atopic status was associated with the production of Th2 cytokines by allergen-stimulated whole blood as might be expected even considering limitations of WBC in contrast to the great part of previous studies that have used isolated cells such as purified lymphocytes and/or dendritic cells or even PBMCs which are enriched leukocytes cultures in either way they can both amplify the signal for cytokine production using protein measurement and also can diminishes that interferences of possible cytokine background. Even though, we also observed in WBC the production of IFN-γ by *D. pteronyssinus*-stimulated PBLs to be more frequent among non-atopic asthmatic children, an unexpected finding. Based on this and other related observations [4-6] we hypothesize that the pathogenesis of non-atopic asthma may involve the production of IFN-γ in response to house dust mite allergen. Recent studies have suggested that house dust mite allergens may directly activate the innate immune response [9] and now we extend this to suggest that house dust mite allergen might also induce Th1-mediated inflammatory responses. Previous works have indicated that non-atopic asthma does not fully fit within the Th1/Th2 shift paradigm [5,6,10] and asthma is also associated to IFN-γ production [5,6]. In fact aforementioned studies found that IFN-γ was being produced together with other Th2-type cytokines [5] and eosinophils on asthmatic subjects [5,6]. Other report had also pointed out that peripheral blood IFN-γ-producing CD4+ and CD8+ T cells from non-atopic asthmatic children were increased in relation to atopic children and inversely associated with eosinophils or airway hyperresponsiveness [9]. However, no study was found related to specific Th1-mediated response to a mite antigen as *D. pteronyssinus*. We also found that in addition to IFN-γ, monocytes are up-regulated in non-atopic asthmatics children. Taken together our results show that IFN-γ may play a role in non-atopic wheezing. In conclusion, this is the first large immunoepidemiological study to report that non-atopic asthma in children is associated with a Th1-cytokine production in response to a mite stimulation (*D. pteronyssinus)*. A better characterization of allergen-induced cytokine profiles is likely to enhance our understanding of the biological mechanisms of asthma and related diseases, and could offer a line of explanation for the high prevalence of non-atopic asthma in Latin America [3,11,12] as a consequence of an environment with a high burden of infectious agents attenuating Th2 mediated phenomena but activating other elements that mediate non-allergic inflammatory pathways.

## Abbreviations

GM: Geometric mean; IFN-γ: Interferon-gamma; IL: Interleukin; PBL: Peripheral blood leukocytes; PBMCs: Peripheral blood mononuclear cells cultures; SPT: Skin prick test; sIgE: Specific immunoglobulin E; WBC: Whole blood cultures.

## Competing interests

All authors declare they have no competing financial interests. This study was funded by The Wellcome Trust, UK, HCPC Latin America Excellence Centre Programme, Ref 072405/Z/03/Z.

## Authors’ contributions

CAF has performed some laboratory assays and wrote the manuscript together with MLB. MLB has coordinated the epidemiological work, planned and revised the manuscript. NMAN coordinated the laboratory work and revised the text. PJC and AAC have suggested analysis and revised the manuscript. LDA and NBS have carried out the statistical analysis. LDA has revised the text. All authors read and approved the final manuscript.
